# Research status and hotspots of oral frailty in older adults: a bibliometric analysis from 2013 to 2024

**DOI:** 10.3389/froh.2025.1533159

**Published:** 2025-07-14

**Authors:** Jiaojiao Wu, Doudou Lin, Weibing Chen, Lili Zhang, Xiangying Shen, Dou Fu, Yinglin Li, Xiaojie Ma, Zhongxiang Cai

**Affiliations:** 1 Department of Geriatrics, Renmin Hospital of Wuhan University, Wuhan, China; 2 Department of Nursing, Renmin Hospital of Wuhan University, Wuhan, China

**Keywords:** older adults, oral frailty, research trends, research hotspots, visual analytics, bibliometric

## Abstract

**Background:**

Oral frailty has emerged as a critical focus in public health due to its strong association with adverse health outcomes in older adults, such as cognitive decline, malnutrition, falls, disability, and mortality. Despite a growing body of research over the past decade, a comprehensive bibliometric analysis of this field remains lacking. This study addresses this gap by providing an overview of the research landscape, highlighting key achievements, identifying emerging trends, and proposing directions for future exploration.

**Methods:**

A bibliometric analysis was conducted on literature related to oral frailty in older adults published between 2013 and 2024, utilizing the Web of Science Core Collection (SCIE and SSCI). The analysis employed CiteSpace, VOSviewer, and the “bibliometrix” R package to visualize and evaluate contributions from countries/regions, organizations, authors, journals and articles. Additionally, references and keyword analyses were performed to identify research patterns and thematic trends.

**Results:**

The bibliometric analysis of 847 articles published from 2013 to 2024 revealed that Japanese scholars contributed the most publications in the field (*n* = 204), representing 24.09% of the total. The cooperation network map revealed the highest intensity of collaboration among researchers from the United Kingdom, the United States and Japan. Watanabe Y and Hirano H, both from Japan, were identified as the most prolific and frequently co-cited authors. The research focuses on the multifactorial mechanisms of oral frailty, comprehensive intervention measures and quality of life. Key research hotspots in the field included tongue pressure, tooth loss, social support, quality of life, health promotion, dental care, and root caries. Emerging research directions may include inflammation, swallowing function and oral function.

**Conclusion:**

Research on oral frailty in older adults has advanced significantly over the past decade, with Japan making particularly notable contributions to the field. The multifactorial mechanisms of oral frailty, multidimensional evaluation methods, and comprehensive intervention strategies are expected to remain central research focuses. Our findings aim to provide researchers with a clearer understanding of trends within this field.

**Systematic Review Registration:**

https://www.crd.york.ac.uk/PROSPERO/view/CRD42024570590, PROSPERO CRD42024570590.

## 1 Introduction

According to the “World Population Prospects 2024” (1) report released by the United Nations Department of Economic and Social Affairs, the global population is projected to reach 8.16 billion in 2024, with the number of individuals aged 60 and over reaching 1.18 billion. The report further forecasts that by 2100, the global population will increase to 10.18 billion, the number of people aged 60 and over will rise to 3.02 billion. The ageing of the global population represents a critical medical and socio-demographic challenge that must be addressed globally (2).

With the global population ageing at an accelerating rate, the health of older adults has become a priority concern. Functional disability is a prominent issue associated with aging, contributing to a significant decline in quality of life among the older adult. Risk factors for functional disability in this population include geriatric syndromes and related conditions, such as frailty (3).

The concept of frailty is widely recognized as a state of increased vulnerability to stressors, resulting from age-related declines in function and reserves across multiple physiological systems (4). In essence, frailty represents a condition where the reserve capacity of various physiological systems has diminished to a point where minor disturbances can lead to significant health issues. A comprehensive meta-analysis encompassing 240 studies from 62 countries and territories, involving a total of 1,755,497 participants over 50 years of age, reported a pooled prevalence of 12% (95% CI = 11%–13%; *n* = 178) for studies using physical frailty measures, compared with 24% (95% CI = 22%–26%; *n* = 71) for the deficit accumulation model (5). Many previous studies have demonstrated that frailty is a multidimensional and comprehensive concept, encompassing physiological, social, and cognitive domains. In recent years, the concept of oral frailty has gained increasing attention. Oral frailty is a concept recently introduced in Japan and presently considered a major determinant of dental and oral health policies. Although oral frailty has not yet been fully conceptualized, it is an important indicator for evaluating declines in oral function, and has increasingly garnered attention. Currently, the most widely used definition of oral frailty, as proposed by the Japan Dental Association in 2020, describes it as a mild decline in oral function, accompanied by declines in physical and mental function, occurring in the reversible stage and the early stage of frailty (6). A systematic review and meta-analysis of 18 studies involving 12,932 older adults revealed that the combined prevalence of oral fragility and preoral frailty was 24% and 57%, respectively. Oral health is a critical component of overall health, and oral frailty is considered an early manifestation of physical frailty. Numerous studies have shown that oral frailty is associated with adverse health outcomes, including overall frailty, sarcopenia, long-term care needs, and premature mortality among community-dwelling older adults (7–9).

Bibliometrics is an emerging method of literature analysis that allows for both quantitative and qualitative assessment of articles in relevant fields, summarizing various characteristics of the included literature, such as countries, institutions, journals, authors, citation references, and keywords, thereby revealing the topics and trends of popular research areas that have been widely applied across numerous disciplines (10–13). In recent years, the number of studies on oral frailty has increased, but bibliometric methods have yet to be employed to analyze and understand the overall landscape of this field. Therefore, using CiteSpace 6.3.3, VOSviewer 1.6.20 and the “bibliometrix” package in R, we analyzed literature in the field of oral frailty among older adults from the Web of Science Core Collection (WoSCC) database, spanning from 2013 to 2024. The analysis aimed to explore the research status, hotspots, and development trends in this field, providing a foundation for future research.

## 2 Methods

### 2.1 Data sources and search strategies

The Web of Science Core Collection (WOSCC) is widely recognized as a premier database for bibliometric analysis due to its extensive literature coverage and academic influence (10, 14–16). It supports data formats compatible with major bibliometric tools, facilitating efficient data analysis and processing (10, 17–23). Given that research on oral frailty in older adults spans both biomedical and social science domains, the Science Citation Index Expanded (SCIE) and the Social Sciences Citation Index (SSCI) within WOSCC were selected as data sources to ensure data comprehensiveness and quality, thereby enhancing the reliability and validity of the bibliometric analysis results.

Based on clinical experience, relevant concepts of oral frailty in older adults, medical subject headings (MeSH) and references to several published studies (24–26), the search strategy of this study was finalized as follows: TI = (“aged*” OR “aging” OR “elder*” OR “senior*” OR “frail older adult” OR “advanced age” OR “geriatric” OR “older adult*” OR “older people” OR “older patient*” OR “older person” OR “older population” OR “older individuals”) AND TI = (“oral frail*” OR “oral health” OR “oral function” OR “oral hypofunction” OR “decrease in oral function” OR “deterioration of oral function” OR “oral health deterioration” OR “decreasing oral function” OR “decline in oral function” OR “rapid oral health deterioration” OR “decline in oral health” OR “oral weakness” OR “oral dysfunction” OR “oral intake” OR “oral hygiene” OR “oral health-related quality of life” OR “tooth loss” OR “number of teeth” OR “masticatory ability” OR “chewing ability” OR “oral diadochokinesis” OR “oral flexibility” OR “tongue pressure” OR “tongue strength” OR “oral motor function” OR “articulation function” OR “chewing difficulty” OR “swallowing difficulty”) NOT [TI = (child* OR student* OR adolescent* OR teenager* OR schoolchildren OR infant* OR mice OR mouse OR rat* OR dog* OR animal* OR birth OR school OR babies OR reproductive OR breastfeeding OR working age)]. The literature search period spanned from January 1, 2013, to July 19, 2024, and was restricted to English-language articles and review articles. A total of 951 articles were initially identified.

### 2.2 Data collection and analysis

**Figure 1:**
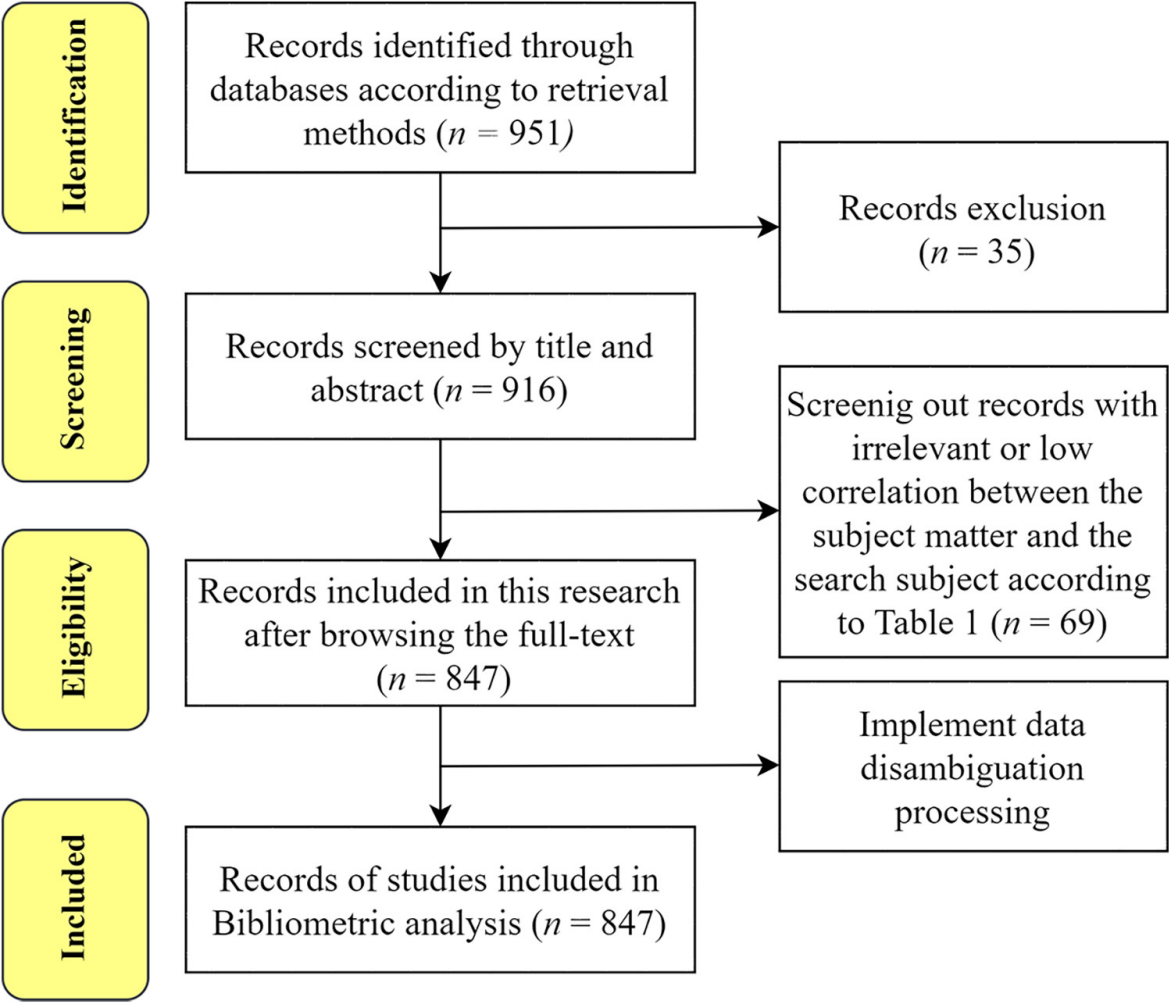
PRISMA flowchart for including studies to review.

Two researchers independently conducted data retrieval and screening, resolving discrepancies through discussion; a third reviewer was consulted when necessary to ensure consistency and reduce subjective bias. The selection process adhered to PRISMA guidelines and comprised four stages: identification, screening, eligibility, and inclusion (27–29). An initial search yielded 951 records. Following the screening criteria outlined in Table 1, non-research items (e.g., book reviews, editorials, corrections, news articles, brief reports, conference abstracts, and commentaries), non-English publications (*n* = 33), and studies unrelated to the research topic were excluded, resulting in 847 publications being retained for analysis. The full screening flowchart is shown in Figure 1. To ensure conceptual precision and topic specificity, we employed an operational definition of “oral frailty.” Drawing on the definition proposed by Tanaka et al. (8), oral frailty is conceptualized as a multidimensional decline in oral function—including deterioration in chewing ability, swallowing, speech, tongue motor function, and oral hygiene—that contributes to adverse health outcomes such as physical frailty, cognitive decline, and increased mortality. Eligible studies were required to either: (1) explicitly use the terms “oral frailty,” “oral hypofunction,” or other related concepts indicating multidimensional oral functional decline among older adults; or (2) focus on diagnostic criteria, health outcomes, or intervention strategies aligned with established clinical definitions of oral frailty. Studies primarily addressing general oral health without reference to frailty-related constructs were excluded. For literature with varying terminology or borderline relevance, inclusion was determined based on whether the study captured key features of oral frailty (e.g., functional decline across multiple oral domains) or adhered to frameworks consistent with the definitions proposed by the Japanese Society of Gerodontology or Tanaka et al. (2018). Full-text review and group consensus (including a third reviewer when needed) were used to ensure methodological rigor and minimize subjective interpretation. This study has been registered with PROSPERO (Protocol number: CRD42024570590). Data were exported in “plain text file” format, including full records and cited references. To enhance consistency and reduce meaningless repetition in the analysis, keywords were standardized by merging synonyms, aliases, and singular/plural forms. Data organization was carried out using Microsoft Excel 2021. Bibliometric analyses were subsequently performed using CiteSpace (version 6.3.3), VOSviewer (version 1.6.20), and the “bibliometrix” package in R (version 4.4.1).

**Table 1:** Inclusion criteria.

Types	Inclusion criteria
Research Content Criteria	Literature with core themes related to oral frailty in older adults, such as focusing on the concept, causes, clinical manifestations, diagnosis, treatment and care, influencing factors, adverse health consequences, and closely related topics like oral frailty assessment tools.
Research Quality Standard	Literature must be at least five pages in length. Reports or short papers under five pages will not be included.
	Literature should include key information elements (e.g., abstract, author information, keyword fields, references). Literature lacking essential information elements will be excluded.

## 3 Result

### 3.1 Scientific output

**Figure 2:**
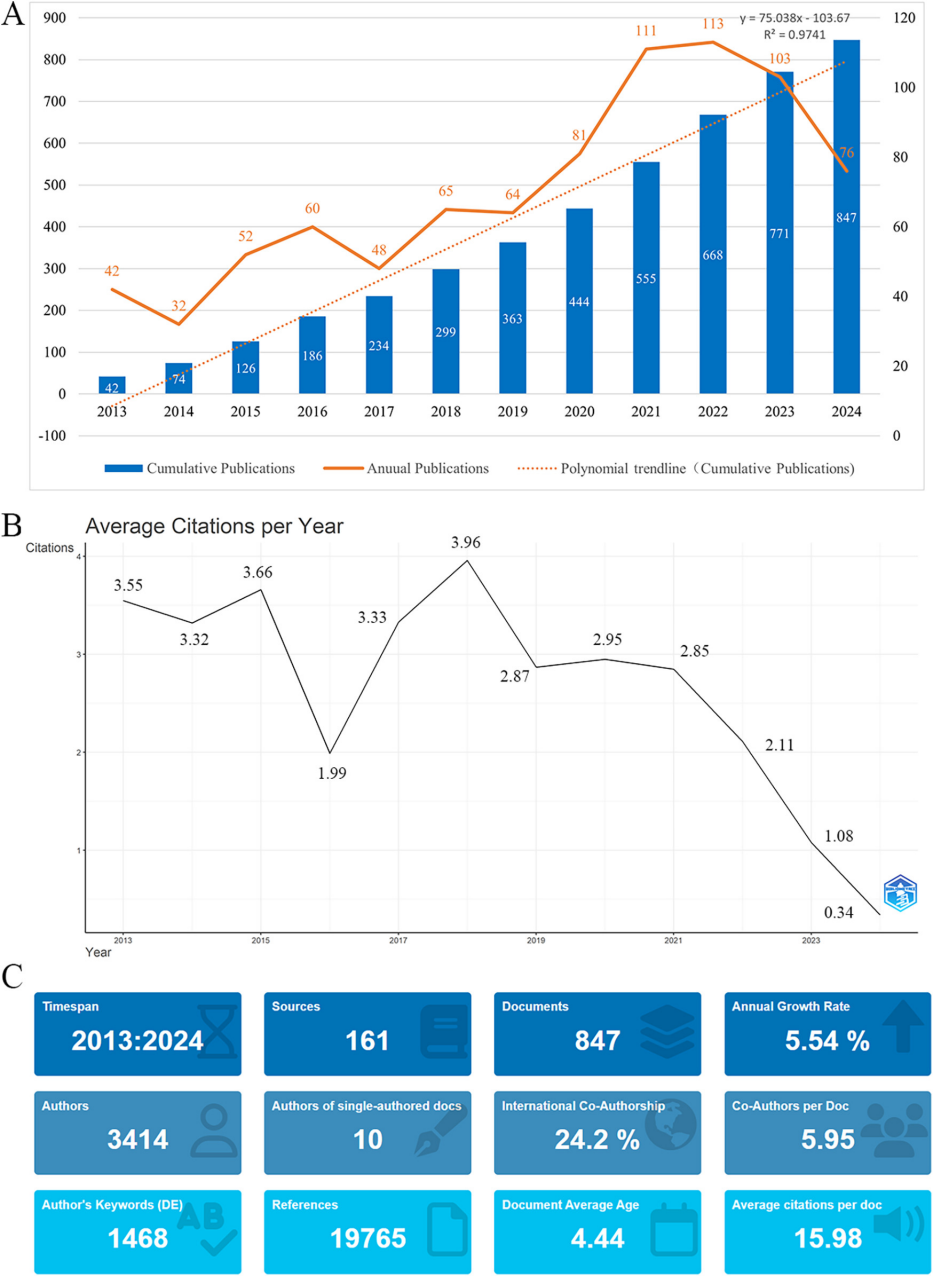
**(A)** Annual and cumulative publication output on oral frailty in older adults from 2013 to 2024. **(B)** Average citations per publication per year from 2013 to 2024. **(C)** Additional statistics on publications about oral frailty in older adults from R bibliometrix.

Our search identified a total of 847 international publications on oral frailty in older adults from January 1, 2013, to July 19, 2024 (Figure 2A). The number of publications grew most significantly between 2020 and 2021, with the highest annual publication volume observed in 2022, reflecting the increasing global interest in this topic. The cumulative number of publications from 2013 to 2024 exhibited a strong linear growth trend, modeled by the equation *y* = 75.038*x* – 103.67 (*R*^2^ = 0.9741). On average, each publication received 2.67 citations per year, peaking at 3.96 citations per publication in 2018 (Figure 2B). The references, journal sources and additional statistical details are shown in Figure 2C.

### 3.2 Countries

**Figure 3:**
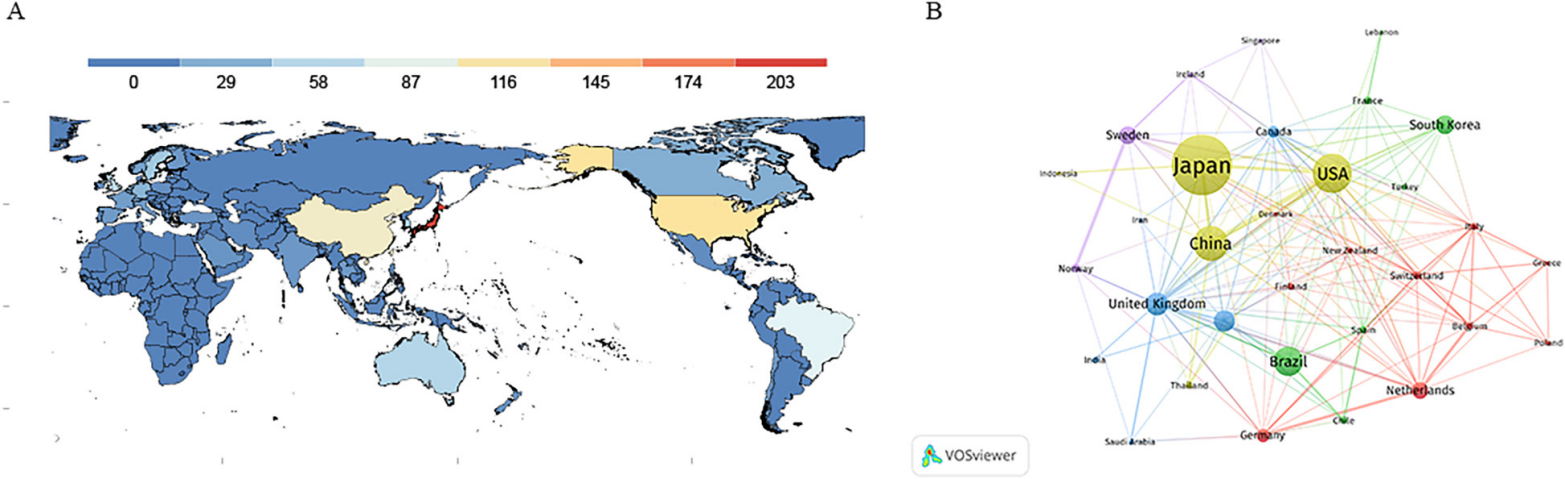
**(A)** World map showing the distribution of research on oral frailty in older adults from 2013 to 2024. **(B)** Cooperation network map of countries from 2013 to 2024.

**Figure 4:**
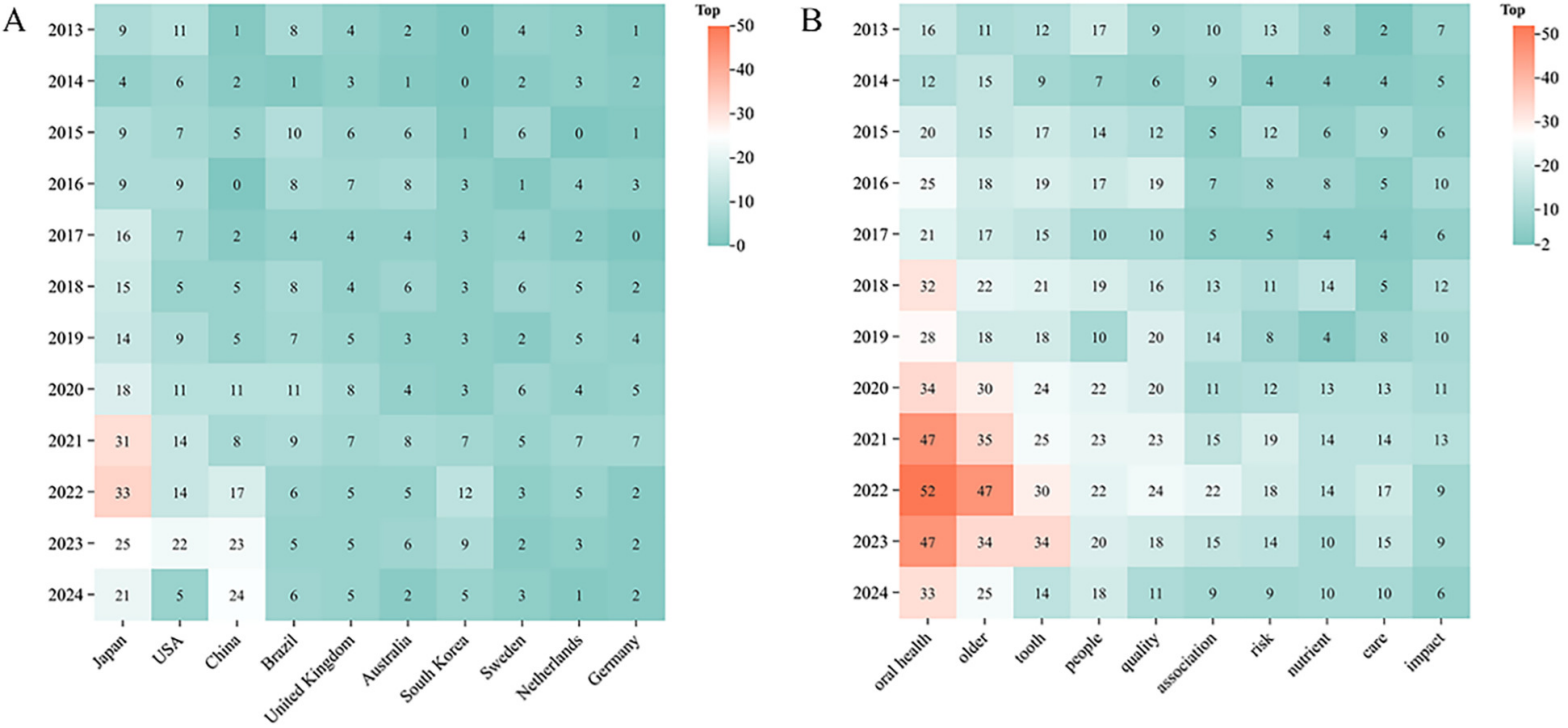
**(A)** Annual fluctuations in academic output for the high-productivity countries (top 10), **(B)** highfrequency keywords (top 10) from 2013 to 2024.

The global distribution of published articles is shown in Figure 3A. A total of 178 countries have contributed to research on oral frailty in older adults. The top 10 countries by publication volume are listed in Table 2. Japan leads with the largest contribution (*n* = 204), accounting for 24.09% of the total publications and receiving 3,812 citations, significantly more than other countries. This is followed by the United States (*n* = 120) and China (*n* = 103). The Netherlands has the highest average citations per publication, at 29.62. Figure 3B illustrates the collaborative relationships between countries. In the network graph, each node represents a country, with node size indicating publication volume and total connection strength reflecting collaboration intensity. The United Kingdom, the United States, and Japan exhibit the highest total connection strengths (84, 84, and 46, respectively), indicating strong international collaboration. Annual publication trends of the top 10 countries are shown in Figure 4A, highlighting Japan's publication peak in 2021/2022.

**Table 2:** Top 10 countries by number of publications (2013–2024).

Rank	Country	Documents	Percentage	Citations	Average citation per paper
1	Japan	204	24.09%	3,812	18.69
2	USA	120	14.17%	1,881	15.68
3	China	103	12.16%	953	9.25
4	Brazil	83	9.80%	1,285	15.48
5	United Kingdom	63	7.44%	1,346	21.37
6	Australia	55	6.49%	794	14.44
7	South Korea	49	5.79%	334	6.82
8	Sweden	44	5.19%	759	17.25
9	Netherlands	42	4.96%	1,244	29.62
10	Germany	31	3.66%	510	16.45

### 3.3 Journal

**Figure 5:**
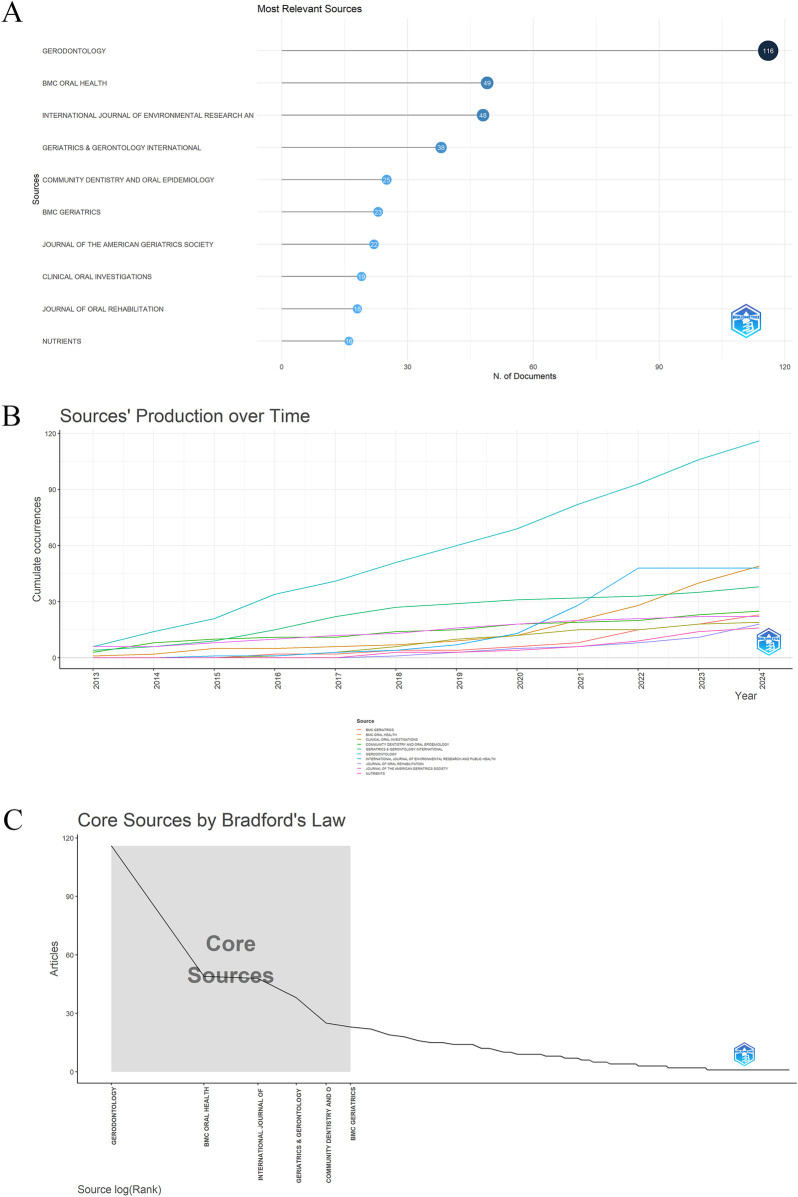
**(A)** Top 10 journals on oral frailty in older adults by publication count. **(B)** Journal output trends within the top 10 from 2013 to 2024. **(C)** Delineation of core and non-core journals according to Bradford's law.

A total of 161 journals worldwide have contributed to research on oral frailty in older adults (Figure 2C). Figure 5A displays the top 10 journals based on the number of publications. From 2013 to 2024, the cumulative journal output has steadily increased (Figure 5B), with *Gerodontology* exhibiting a growth rate significantly higher than that of other journals. Among the top 10 journals, *Gerodontology* leads with 116 publications, followed by *BMC Oral Health* (*n* = 49) and the *International Journal of Environmental Research and Public Health* (*n* = 48). According to Bradford's law (30), these 161 journals are divided into three zones, with six core source journals in Zone 1 (Figure 5C; Table 3).

**Table 3:** According to Bradford's law, the 161 journals were classified into zones 1–3.

Zone	No. of journals	No. of publications	Percentage
1	6	299	35.3%
2	22	271	32.0%
3	133	277	32.7%
Total	161	847	100.0%

### 3.4 Author analysis

**Figure 6:**
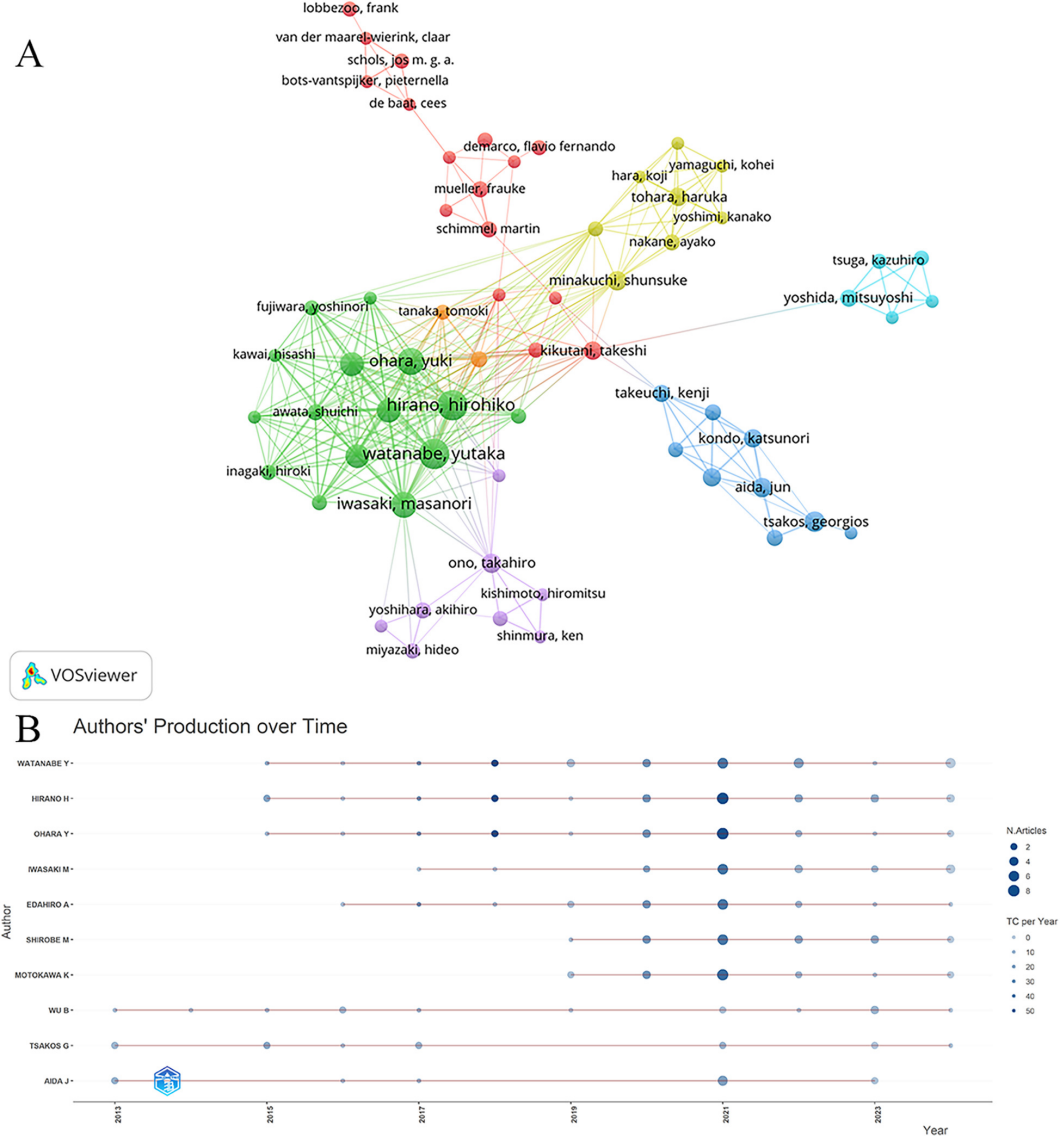
Analysis of the cited authors. **(A)** Author co-authorship network map. Nodes of different colors represent groups of authors with close collaborative relationships. **(B)** Authors’ production over time. Node size indicates the number of documents, and color intensity represents the average number of citations per year.

Analyzing the authors in this field provides insight into the key scholars and primary contributors to research on oral frailty among older adults. Data analysis reveals a total of 3,414 authors in this domain. Table 4 presents the top 10 authors based on publication count from 2013 to 2024. Key metrics such as the H-index, G-index, M-index, total citations, and publication years are provided for each author. The top three authors are Watanabe Y (*n* = 28), Hirano H (*n* = 27), and Ohara Y (*n* = 22). Watanabe Y and Hirano H, both affiliated with the Tokyo Metropolitan Institute of Gerontology in Tokyo, Japan, lead in publication count, H-index, and G-index, reflecting their substantial impact on the field. The H-index, a metric that measures a scholar's academic impact based on both publication quantity and citation count, reflects the scholarly influence of these authors, with higher values indicating greater impact. The G-index, another metric used to assess a researcher's influence, is calculated by dividing the number of citations of their most influential papers by the total number of citations (31). The three most-cited authors in the field of oral frailty among older adults are Hirano H (1,194 citations), Watanabe Y (1,083 citations), and Ohara Y (1,038 citations). Figure 6A illustrates the collaboration network among authors. Using VOSviewer, the minimum number of publications per author was set to five, resulting in a total of 84 authors grouped into seven clusters. Each cluster represents distinct research directions or regionally based research teams within the field. For example, the green cluster centers around Watanabe Y, while the red and blue clusters signify other collaborative networks. The number and thickness of the connecting lines indicate the strength of collaboration between authors. Core authors, such as Hirano H and Watanabe Y, are linked to more nodes, highlighting their central role in the field. Additionally, some lines connect clusters of different colors, indicating cross-group collaborations. Additionally, Figure 6B visualizes the publication output and citation impact of influential authors in oral frailty research over time. The timeline format reveals that authors like Watanabe Y, Hirano H, and Ohara Y exhibit sustained scientific influence, marked by consistent publication records and periods of notably high average citation rates. This sustained influence, evident throughout the 2013–2024 period, underscores their significant contributions to the field's evolving knowledge base.

**Table 4:** Top 10 authors in terms of publications from 2013 to 2024.

Rank	Author	H-index	G-index	M-index	Publications	Total citation	PY_start
1	Watanabe Y	16	28	1.455	28	1,083	2015
2	Hirano H	17	27	1.545	27	1,194	2015
3	Ohara Y	16	22	1.455	22	1,038	2015
4	Iwasaki M	11	18	1.222	19	326	2017
5	Edahiro A	13	18	1.300	18	579	2016
6	Shirobe M	12	18	1.714	18	373	2019
7	Motokawa K	12	17	1.714	17	355	2019
8	Wu B	8	14	0.615	14	312	2013
9	Tsakos G	9	12	0.692	12	376	2013
10	Aida J	8	11	0.615	11	302	2013

### 3.5 References burst test

**Figure 7:**
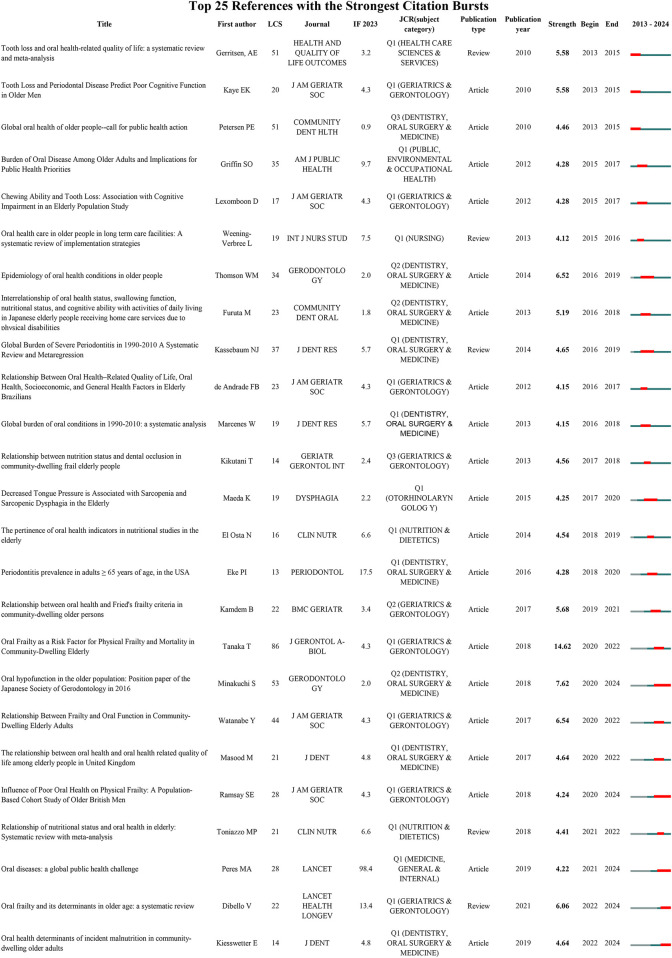
Top 25 references with the strongest citation bursts, including information on first authors, citations, journals, and publication types, arranged in ascending order based on burst time. LCS: Local citation score.

The 19,765 references constitute the foundational knowledge base of this field (Figure 2C). References with high burst intensity often represent substantial scientific contributions and have a considerable influence on subsequent research. Figure 7 lists the top 25 references with the strongest citation bursts. Red bars indicate burst intensity, duration, and time period. Leading this list is the 2018 study by Tanaka T et al., titled *Oral Frailty as a Risk Factor for Physical Frailty and Mortality in Community-Dwelling Older Adult* (strength = 14.62), which has received significant attention in the research community. This is followed by the 2018 publication by Minakuchi S et al., *Oral Hypofunction in the Older Population: Position Paper of the Japanese Society of Gerodontology in 2016* (strength = 7.62), and the 2017 study by Watanabe Y et al., *Relationship Between Frailty and Oral Function in Community-Dwelling Older Adults* (strength = 6.54). Notably, Minakuchi S et al.'s 2018 paper continues to experience citation bursts, underscoring its sustained relevance and impact within the field.

### 3.6 Keyword visualization and burst test

**Figure 8:**
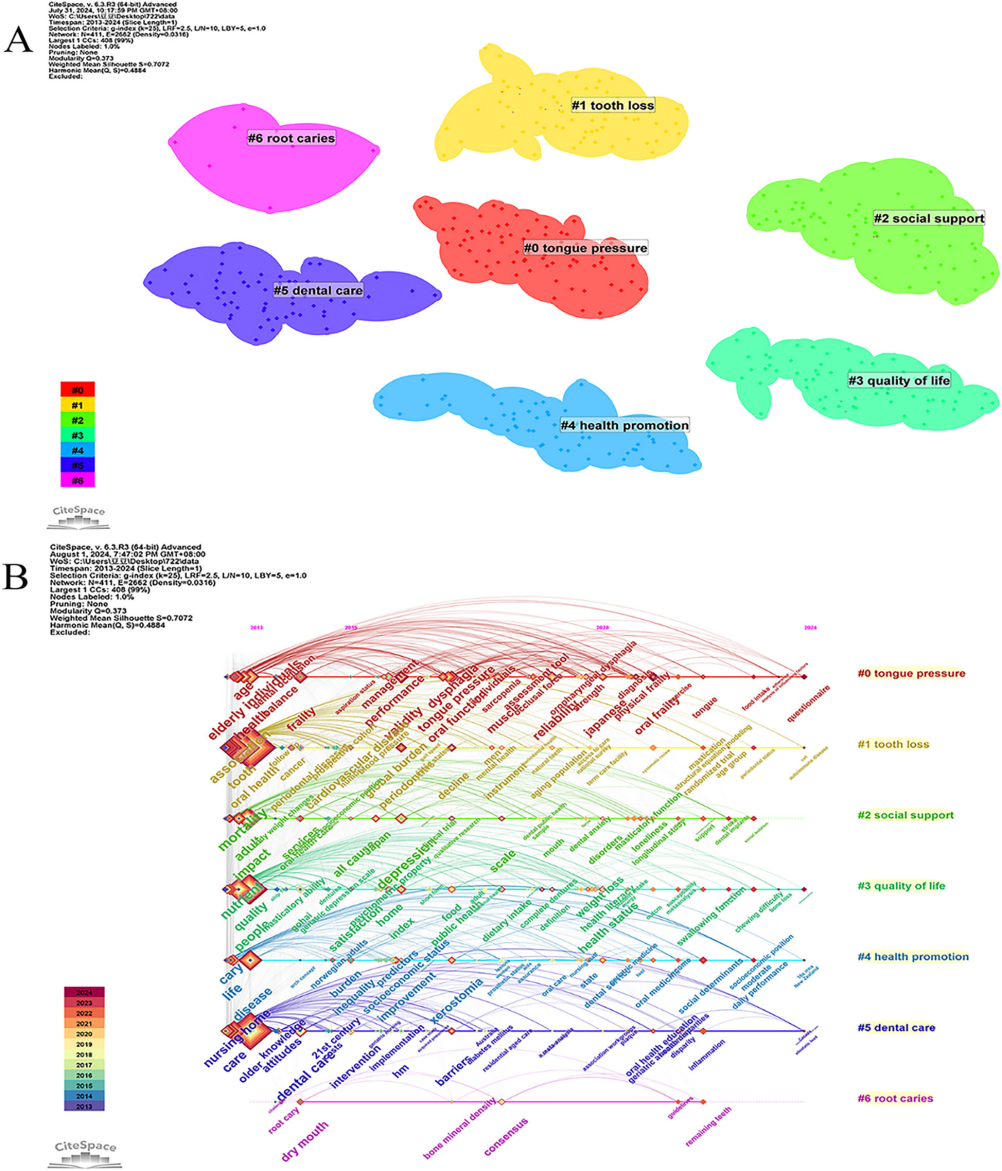
Keywords analysis. **(A)** Map of keywords related to oral frailty in older adults; **(B)** A timeline view of keywords.

**Figure 9:**
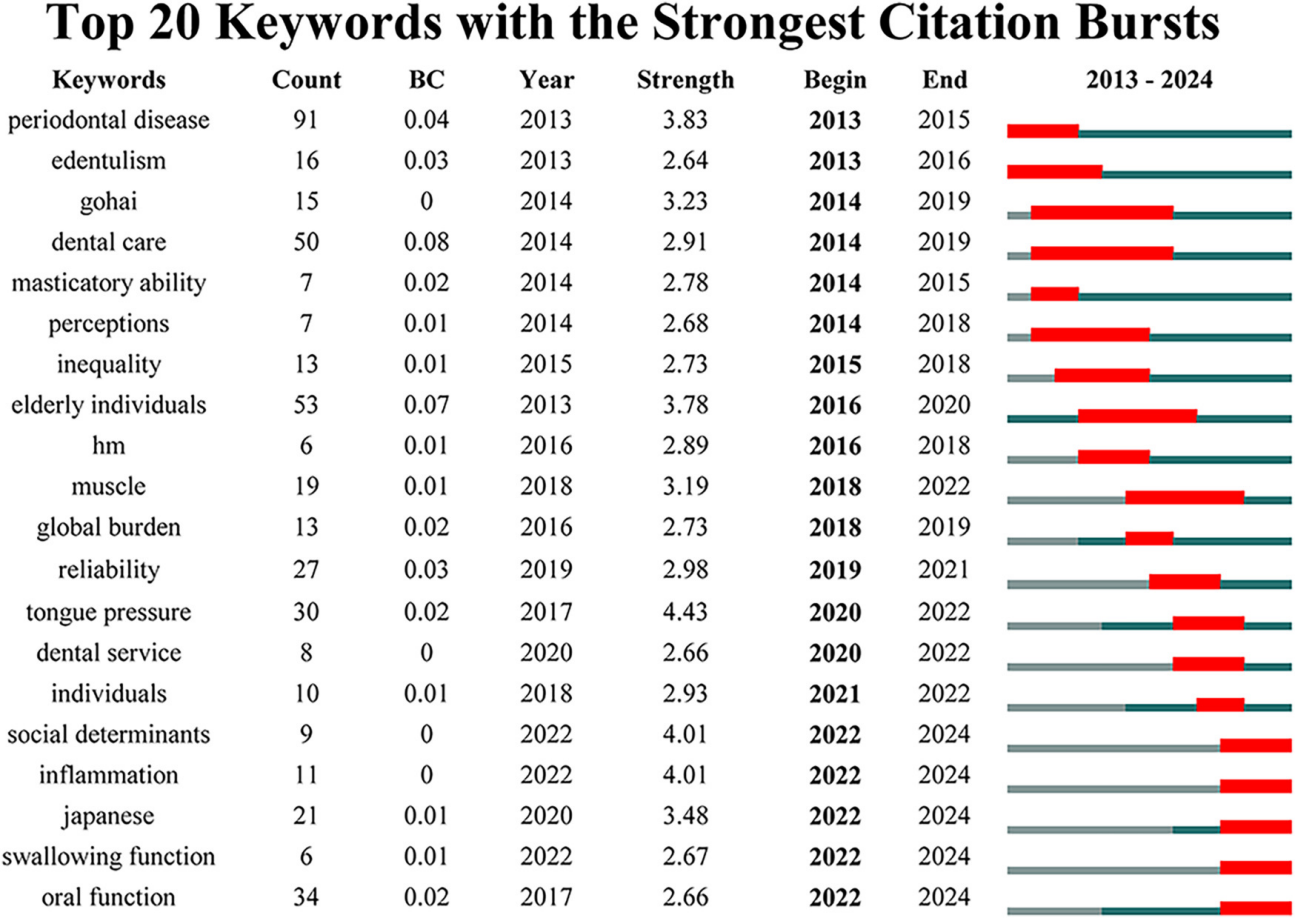
Top 20 keywords with the strongest citation bursts, including information on count, BC(betweenness centrality), and year, arranged in ascending order based on burst time.

A total of 411 keywords were extracted from the 847 publications, with the top 10 high centrality and high-frequency keywords listed in Table 5. These keywords experienced peaks in research activity between 2021 and 2023 (Figure 4B). The three most frequently occurring keywords were “oral health” (*n* = 367), “older” (*n* = 287), and “tooth” (*n* = 238). Keyword clustering and burst detection analyses were conducted using CiteSpace(version 6.3.3). The key parameter settings were as follows: time span = 2013–2024; time slicing = 1 year; g-index (*k* = 25); LRF = 2.5; LBY = 5; *e* = 1.0; and no pruning was applied. Cluster labeling was automatically generated by the software using the log-likelihood ratio (LLR) algorithm based on high-frequency terms within each cluster. The extracted keywords were grouped into seven thematic clusters (Figure 8A): including “tongue pressure,” “tooth loss,” “social support,” “quality of life,” “health promotion,” “dental care,” and “root caries.” The quality of clustering was validated using two structural metrics: the modularity score (*Q* = 0.372) and the silhouette score (*S* = 0.7072). A Q value above 0.3 suggests a significant modular structure, while a silhouette score above 0.7 reflects high intra-cluster homogeneity and good separation between clusters. The top three clusters identified were 0# “tongue pressure,” 1# “tooth loss,” and 2# “social support,” suggesting that these themes are the most prominent research areas in oral frailty among older adults. The keyword timeline visualization illustrates the evolution of research focus (Figure 8B), revealing a transition from basic oral health status to a deeper exploration of the multifactorial mechanisms underlying oral frailty, multidimensional assessment methodologies, and comprehensive intervention strategies. To identify emerging trends, keyword burst detection was also conducted in CiteSpace with a burst sensitivity parameter *γ* = 0.5 and a minimum burst duration of 2 years. Burst detection is instrumental in capturing short-term surges of interest in specific topics. In Figure 9, the blue bars denote the full time span, while red bars mark the burst periods. The top 20 keywords with the highest burst strength are presented, with “tongue pressure,” “social determinants,” and “inflammation” ranking as the top three. These keywords likely constitute the core of contemporary research on oral frailty in older adults. Moreover, in recent years, keywords such as “social determinants,” “inflammation,” “swallowing function,” and “oral function” have emerged as significant research directions.

**Table 5:** Top 10 keywords by betweenness centrality and frequency.

Rank	Keyword	Centrality	Keyword	Frequency
1	Dental care	0.08	Oral health	367
2	Dementia	0.07	Older	287
3	Older adult individuals	0.07	Tooth	238
4	Dental caries	0.07	People	199
5	Chewing ability	0.07	Quality	188
6	Life	0.07	Association	136
7	Association	0.06	Risk	133
8	Nutrient	0.06	Nutrient	109
9	Prevalence	0.06	Care	106
10	Health	0.06	Impact	104

The left column presents the top 10 keywords ranked by betweenness centrality, while the right column shows those ranked by frequency.

## 4 Discussion

### 4.1 General information

This study provides a comprehensive bibliometric and visual analysis of research on oral frailty in older adults from 2013 to 2024, based on data from the Web of Science Core Collection. The analysis demonstrates significant growth in research output, particularly during the period from 2019 to 2021, indicating a heightened scholarly interest in this field. While the number of publications has slightly declined since 2021, this timeframe underscores the recognition of oral frailty as an emerging and significant research topic within gerontology.

Our findings reveal a global research landscape with Japan at the forefront of oral frailty studies, contributing over a quarter (24.09%) of the total publications, followed by the USA and China. Japan's prominence in this field aligns with its foundational role, as Japanese scholars were the first to introduce the concept of oral frailty. The surge in Japanese publications during 2021/2022 underscores the country's pivotal influence in shaping both the volume and quality of research contributions. This substantial output reflects Japan's longstanding focus on geriatric healthcare and its recognition of oral health as a critical component of healthy aging. Notably, the Netherlands, while contributing fewer publications, boasts a high average citation rate per paper (29.62), suggesting that their research is both high-quality and impactful. Conversely, while China has made substantial contributions in terms of publication volume, its relatively low citation rate reflects not only opportunities to enhance the quality and global impact of its research, but also contextual factors worth acknowledging. As highlighted by reviewers, the recency of China's research output in this field (with a significant portion of studies published in the latter half of our 2018–2024 analysis window) limits citation accumulation time. Additionally, potential language - related citation practices (e.g., dissemination in Chinese -language journals or delayed integration into global academic databases) may also influence these metrics.

International collaboration plays a crucial role in advancing this field, with the strongest partnerships observed between the United Kingdom, the United States, and Japan. These high-strength collaborations indicate that these nations not only contribute substantially to research output but also drive knowledge exchange and resource sharing, both vital for progress in this field. Beyond the dominant UK-US-Japan collaborative axis, the regional landscape of collaboration networks reveals smaller, less integrated clusters in Western Europe, such as the fragmented partnerships among the Netherlands, Germany, and France, as well as peripheral subnetworks in regions like Ireland and Iceland. These marginalized groups exhibit sparse connections with the global core network, highlighting gaps in regional integration. By acknowledging both the dominant collaborative patterns and these underrepresented clusters, we achieve a more comprehensive understanding of the global research ecosystem.

Journal analysis indicates that, from 2013 to 2024, a total of 161 journals contributed to relevant publications, with the number of journals in this field increasing over time (Figure 5A). Of these, the journal *Gerodontology* ranked first in the number of publications (*n* = 116), establishing its leading position in the field. As a journal specializing in the oral health of aging populations, *Gerodontology* focuses on topics such as dental care, oral health promotion, and the relationship between oral function and systemic health in older adults. They were followed by *BMC Oral Health* (*n* = 49) and the *International Journal of Environmental Research and Public Health* (*n* = 48). The output of the top ten journals has shown a steady upward trend (Figure 5B), indicating increasing academic interest and investment in understanding oral frailty in old age, likely driven by the global aging population and the need to improve oral care in old age. According to Bradford's Law, 161 journals are classified into zones 1–3, of which six core journals are classified into zones 1. This division not only reflects the number of journals and concentration of publications, but also highlights the key role of core journals in advancing research in the field. The remaining papers were distributed in Region II (22 journals, 32.0%) and Region III (133 journals, 32.7%). This distribution underscores that the concentration of high-impact research in a small number of journals, especially those in Region 1, is the primary source of important contributions in the field.

Author analysis revealed that a total of 3,414 scholars contributed to research on oral frailty in older adults. Among them, Watanabe Y, Hirano H, and Ohara Y are the top three contributors, with 28, 27, and 22 publications, respectively. These scholars can be considered the core researchers in this field, as their H-index and G-index demonstrate significant academic influence. All three are affiliated with the Tokyo Metropolitan Institute of Gerontology, and their collaborative work has yielded several papers addressing key aspects of oral frailty, including the development of assessment tools and their application to the older adult population. Their research explores the association between oral frailty and physical frailty, cognitive function, malnutrition, and mortality, as well as the relationship between chewing ability, sarcopenia, and Masseter muscle tension in community-dwelling older adults. These contributions have advanced our understanding of the complex interplay between oral health and overall well-being in aging populations. The cooperative network diagram (Figure 6A) illustrates the collaborative relationships among these authors, highlighting six distinct, color-coded research groups. This collaboration not only fosters the dissemination of knowledge but also enriches the diversity of research perspectives in this field.

Reference burst analysis highlights pivotal contributions that have shaped the current understanding of oral frailty in older adults. The 2018 study by Tanaka T et al., *Oral Frailty as a Risk Factor for Physical Frailty and Mortality in Community-Dwelling Older Adult*, stands out as the most highly cited reference, with a burst strength of 14.62. This work significantly advanced the field by linking oral frailty to physical frailty and mortality, identifying oral frailty as a critical risk factor for broader health outcomes in older adults. Its sustained citation bursts underscore its lasting impact, both theoretically and practically, in shaping subsequent research on the multifaceted effects of oral health on aging. Minakuchi et al.'s 2018 position paper, *Oral Hypofunction in the Older Population*, remains a highly cited and ongoing research hotspot. Its sustained citation burst underscores the increasing recognition of oral dysfunction as a core element of health management in older adults. Similarly, Watanabe et al.'s 2017 study, *Epidemiology of Oral Health Conditions in Older People*, further enriched understanding of the prevalence and impact of oral health issues in aging populations. The continued surge in citations of recent studies and reviews highlights the growing emphasis on integrating oral health into overall health assessments and interventions, particularly for older adults. This trend suggests that oral health is increasingly recognized not only as a localized issue but also as a key factor influencing broader health outcomes, including physical function and nutritional status. The rising focus on oral frailty and its determinants reflects a shift towards developing targeted strategies for prevention and management.

### 4.2 Description of future trends

Keywords serve as highly concise summaries of the literature, and cluster analysis can classify them according to its algorithms. This keyword analysis provides valuable insights into the evolving research landscape of oral frailty in older adults. The analysis reveals a field transitioning from a focus on basic oral health to a more nuanced understanding of the multifaceted nature of oral frailty and its impact on overall health and well-being. The top ten keywords, including “oral health,” “older adult,” and “tooth,” confirm the core themes of the field (Table 5). However, a deeper analysis of keyword clusters and time-series data reveals a more complex picture. According to the cluster analysis, categories such as “tongue pressure”, “root caries”, “tooth loss”, “dental care”, “social support”, “health promotion”, and “quality” emerge as particularly noteworthy (Figure 8A). By combining keywords with clustering analysis, this study identifies three research hotspots in this field, as follows.

1. Multi-factor mechanism of oral frailty: Oral frailty, as a dimension of frailty, is influenced by multiple factors. In his research, Tanaka et al. (8) explored the operational definition of oral frailty. He identified six evaluation indicators:the number of natural teeth, chewing ability, oral articulation function, tongue pressure, subjective difficulty in eating tough foods and subjective difficulty in swallowing as evaluation indicators of oral frailty. Cluster analysis in this study revealed that tongue pressure and tooth loss emerged as prominent factors (Figure 8A). These findings align with Tanaka et al.'s framework, highlighting two key aspects among the six indicators of oral frailty.Tongue pressure is the pressure created when the tongue makes contact with the hard palate during swallowing and it is used to assess the strength of the tongue muscles (32). Adequate tongue pressure is essential for maintaining swallowing function. Tooth loss can seriously impair an individual's oral function. Studies (33–36) have demonstrated a strong association between tooth loss and decreased oral health quality of life, reduced diet quality, compromised nutritional status, and even mortality, aligning with the adverse effects of oral frailty.
2. Effective interventions: Cluster analysis showed categories such as “root caries”, “tooth loss”, “dental care”, “social support” and“health promotion,” suggesting potential effective interventions. Oral basic health status is an important factor affecting oral frailty. Root caries and tooth loss are the most prevalent oral health issues in older adults. Advances in the prevention and treatment of caries and periodontal diseases have resulted in improved oral health and better tooth retention in the adult population (37). For tooth loss, studies (38) have shown that the prevalence of oral frailty decreases with the number of teeth, emphasizing the importance of timely tooth replacement for maintaining oral function. A study examining changes in oral, chewing, and dietary awareness demonstrated that monthly gatherings at a community center to learn about oral health and nutrition, while consuming a “chew” textured lunch with appropriate nutrients, improved participants’ attitudes toward chewing, oral health, and eating, as well as their overall well-being. This study demonstrates the importance of dental care, health promotion, and social support in maintaining oral health (39). Oral health literacy is closely related to oral health status, making health promotion initiatives, such as oral health knowledge dissemination, particularly important (40). The psychological pathway, specifically the impact of oral health on self-esteem and depression, is one of the mechanisms underlying the links between vulnerability and different health conditions (41). Studies have shown that depressive symptoms or a depressed mood are risk factors for oral frailty. Addressing the negative emotions of older adults and improving depression through psychological intervention are important ways to reduce the occurrence of oral frailty. It is important to note that the importance of social support factors in maintaining oral function is often overlooked (42–45). Several studies have shown that eating alone and social withdrawal are independent risk factors for oral frailty. Therefore, in addition to oral function exercises, social support plays a crucial role in maintaining the oral function of older adults (44, 46). A potential approach involves organizing older adults to eat together or gather (47, 48).
3. Quality of life: Numerous studies have focused on the quality of life of older adults, highlighting the significant concern regarding the impact of oral frailty on their daily lives. The effect of oral frailty on quality of life can be measured through Oral Health-Related Quality of Life (OHRQoL), a critical indicator in dental practice and research. OHRQoL offers a comprehensive assessment of how oral health issues affect physical, psychological, and social functioning. It serves as a multidimensional measure of oral health, encompassing physical and mental health, social relationships, well-being, and satisfaction (49). Oral health is an integral component of overall health, influencing individuals’ physical and mental well-being as well as their social interactions (50). The detrimental impact of oral frailty on OHRQoL is well-established. Existing studies have thoroughly examined the risk factors associated with oral frailty, providing potential pathways to improve OHRQoL. While improving oral frailty is expected to enhance OHRQoL, direct evidence remains limited. Future research should focus on developing interventions to reverse oral frailty, ultimately improving OHRQoL for older adults (51, 52).

Keyword outbreak can provide insight into research hotspots and trends in a certain field. Figure 9 shows the top 20 keywords with burst intensity, combining keywords and cluster analysis, three research frontiers in this field are summarized.

1. Inflammation: The burst analysis (Figure 9) identifies “inflammation” as key emerging research areas. This frontier builds directly on earlier findings in our hotspot analysis, which highlighted periodontal disease as a dominant risk factor for oral frailty (53). The growing prominence of “inflammation” in relation to oral frailty is particularly significant, highlighting the increasing recognition of its role in the pathogenesis and progression of oral frailty. While inflammation is increasingly linked to oral frailty, current evidence remains inconsistent in establishing direct causality, with cross-sectional studies often outweighing longitudinal data (54). Frailty and various oral health conditions can be interconnected through multiple potential mechanisms, including nutritional (the impact of dentition on nutritional status), biological (association with chronic inflammation), and psychological (the effect of oral health on self-esteem and depression) pathways (41). Studies have shown that periodontal disease is an important risk factor for oral frailty, with a 6-year follow-up study suggesting that severe periodontitis is a significant risk factor for new oral frailty in older adults within community settings (53). Periodontal disease refers to diseases affecting the supporting tissues of the teeth (periodontal tissue), including gum disease, which affects only the gum tissue, and periodontitis, which involves deeper periodontal tissues (periodontal membrane, alveolar bone, cementum). Periodontal disease is common and is a leading cause of tooth loss in adults, in addition to being a major oral condition that impacts both dental and overall health (54). The early symptoms of periodontal disease often go unnoticed, leading to chronic infections and repeated inflammation, which not only impair the oral masticatory system but also significantly affect general health. This aligns with a systematic review on immune aging (55), which demonstrated age-related declines in neutrophil function and elevated pro-inflammatory mediators (e.g., IL-1β, IL-6) in periodontitis. Notably, while cross-sectional studies associate periodontal disease with frailty markers, longitudinal evidence remains conflicting. For example, a systematic review (55) of five longitudinal cohorts found strong links between tooth loss/masticatory function and frailty but highlighted heterogeneity in periodontitis-frailty associations due to varying definitions and follow-up durations. Additionally, a narrative review (56) emphasized that most studies cannot distinguish correlation from causation, as periodontal disease may co-occur with confounders (e.g., smoking, diabetes). Longitudinal studies controlling for baseline inflammation and lifestyle factors are needed to establish temporal relationships. Understanding the role of inflammation in the development of oral frailty is critical for advancing our knowledge of frailty in individuals and may provide targets for future clinical interventions. However, current evidence is insufficient to confirm inflammation as a direct driver of frailty. Timely treatment of periodontal diseases and maintenance of oral hygiene can have a positive impact on reducing oral frailty. Future research should prioritize long-term longitudinal designs and standardized periodontal assessments to clarify causal pathways.
2. Social determinants: In addition to inflammatory and psychological factors, social determinants including the emerging concept of “social frailty” play a crucial role in the development of oral frailty. This aligns with our earlier hotspot analysis on “social support,” which identified social disengagement as a recurrent theme in oral frailty research. Social frailty, similar to physical and psychological vulnerability, represents a component of overall frailty but remains less understood compared to physical frailty, with no universally accepted definition to date. It underscores the importance of social factors (e.g., social engagement, support networks) in maintaining health, emphasizing that inadequate social resources can independently increase vulnerability to adverse health outcomes (57). A cross-sectional study in Japan demonstrated that eating alone was significantly associated with oral frailty, suggesting that oral health is linked not only to nutritional status but also to social functioning (44). A longitudinal study in Japan further revealed the interaction between social withdrawal and poor oral health in older adults (46). Additionally, research has shown a relationship between social participation and oral frailty, emphasizing the importance of social determinants in this context (56). Social withdrawal and a reduced social network, often resulting from physical decline and retirement, are common phenomena in the older adult population. A meta-analysis indicated a high prevalence of social vulnerability among older adults, with a total prevalence of 47.3% among hospitalized individuals and 18.8% among those in community settings (58). Therefore, governments should implement policies and provide designated spaces to encourage social interaction among older adults, helping them maintain social functions. Furthermore, other social factors, such as income, education level, and social support, also influence oral frailty. It is essential to screen for oral frailty in populations at risk and implement early, targeted interventions.
3. Oral function: The emergence of “swallowing function” and “oral function” as potential new research directions underscores the critical link between oral health and overall health outcomes in older adults. This directly extends prior hotspot findings on “oral functional assessments” (e.g., tongue pressure, masticatory function) and aligns with existing interventions like oral training programs (59, 60).The essence of the concept of oral frailty is the decline of oral function in the maxillofacial region, emphasizing the importance of oral function and swallowing function for individuals with oral frailty (26). Reversing or delaying functional decline and maintaining existing function are key research goals for the future. As the main vulnerable population, maintaining existing functions is essential for the health of older adults. A randomized controlled trial in Japan applied an oral frailty measures program to community-living older adults, which mainly consisted of oral practice, mouth opening training, tongue pressure training, prosodic training, and chewing training (59). The results revealed significant improvements in articulatory oral motor skills and tongue pressure in the intervention group. In another randomized controlled trial in Japan (60) two groups were compared: one received a paper-based oral function exercise program, where participants followed functional exercises through pictures, while the other used a tablet-based program with video instructions. Both groups participated in the intervention for four weeks, with three sessions per week. The study showed improvements in oral function in both groups, particularly in maximum chewing ability, maximum tongue pressure, and oral diastolic movement. Most existing intervention studies focus on oral function exercises for older adults, including pronunciation exercises, oral practice, mouth opening training, tongue pressure training, prosodic training, chewing training, and tongue and lip motor functions (oral diastolic exercises). These efforts aim to maximize the functional capacity of individuals with oral frailty and align with current research trends.

### 4.3 Recommendations

This study employed a systematic bibliometric approach to analyze research on oral frailty in older adults over the past decade, covering publication trends, country/regional distribution, active organizations, prolific authors, core journals, research hotspots, and emerging trends. These findings provide valuable insights for scientific researchers, offering an overview of the current state and future directions of oral frailty research, as well as informing decision-making for selecting and designing clinical practice interventions. Future research should explore the following areas in greater depth. First, further exploration of the mechanisms of oral frailty is crucial, including understanding its causes and related factors, to identify additional potential intervention targets. Second, expanding the scope of screening to better identify the target population for oral frailty interventions is essential. Finally, conducting more intervention studies to explore a wider range of systematic and diversified approaches is essential for advancing oral frailty management. Oral frailty is closely linked to the overall health and well-being of older adults. Strengthening oral frailty management is crucial for promoting the physical and mental health of older adults, contributing to the achievement of healthy aging.

## 5 Conclusion

This bibliometric study, covering research on oral frailty in older adults from 2013 to 2024, highlights key trends and emerging hotspots in the field. Our analysis identifies Japan as a leading contributor, with a significant focus on exploring the impact of oral frailty on overall health and well-being. Over time, the research landscape has shifted from a primary focus on basic oral health to a deeper understanding of the multifaceted nature of oral frailty and its complex interactions with various health outcomes. Key themes have emerged, including the significance of tongue pressure, tooth loss, and social support, highlighting a growing recognition of the multidimensional aspects of oral frailty in older adults. These insights can inform policymakers and clinicians by identifying priority areas for intervention development, such as targeting social support networks or oral functional assessments, to address oral frailty holistically in aging populations.

## 6 Limitation and future research

First, this study was limited to the Web of Science Core Collection and English-language publications, which may have excluded relevant studies indexed in other databases (e.g., Scopus, PubMed) or published in other languages. This is particularly pertinent in the context of oral frailty, where a substantial body of research—especially from Japan—may be published in Japanese-language journals not indexed in WoSCC. These restrictions may introduce database and language bias, thereby limiting the comprehensiveness and global generalizability of our findings. Second, as oral frailty remains an emerging field, our analysis offers only a cross-sectional snapshot of the evolving research landscape. Third, the relatively small number of intervention-focused studies identified limits our ability to draw robust conclusions regarding effective strategies for managing oral frailty. To address these limitations, future research should adopt a more inclusive and longitudinal approach—integrating multiple databases, considering non-English literature, and capturing trends across time and disciplines—to provide a more comprehensive and globally representative view of this multidisciplinary domain.

## Data Availability

The raw data supporting the conclusions of this article will be made available by the authors, without undue reservation.
